# Editorial: Cell models and preclinical validation of immune-mediating biological therapies

**DOI:** 10.3389/fimmu.2026.1906947

**Published:** 2026-06-26

**Authors:** Alja Zottel, Neja Šamec, Boris Gole, Ivana Jovčevska

**Affiliations:** 1 Center for Human Molecular Genetics and Pharmacogenomics, Faculty of Medicine, University of Maribor, Maribor, Slovenia; 2 Centre for Functional Genomics and Bio-Chips, Institute of Biochemistry and Molecular Genetics, Faculty of Medicine, University of Ljubljana, Ljubljana, Slovenia; 3 Clinical Institute of Special Laboratory Diagnostics, University Children’s Hospital, University Medical Centre Ljubljana, Ljubljana, Slovenia

**Keywords:** 3D cultures, biomarkers, drug development, immunotherapy, organoids, patient-derived models, translational research, tumor microenvironment – TME

Biological drugs such as monoclonal antibodies, vaccines, recombinant and fusion proteins, as well as cell and gene therapies have a greatly improved the treatment of cancer, autoimmune diseases, and other chronic conditions. However, many patients do not respond well to these therapies or develop resistance over time. This emphasizes the need for design of novel therapeutic strategies, or to identify reliable biomarkers for prediction of treatment response. In this regard, development of three-dimensional (3D) cell cultures, co-cultures and organ-on-chip systems, which better mimic *in vivo* conditions, offers better experimental models for drug testing and therapy evaluation, while decreasing the need for animal experimentation. Yet, translation of these complex *in vitro* models into clinical settings remains a challenge.

The goal of the Research Topic “*Cell Models and Preclinical Validation of Immune-Mediating Biological Therapies*” is to collect articles focusing on experimental models and translational approaches that can improve our understanding of immune related therapies. Even though the published papers are not focusing on any single disease, their common denominator is the development of more physiologically relevant disease models for studying disease mechanisms, safety and therapeutic efficacy. Such examples are the 3D models that better mimic tumor microenvironment and are therefore more suitable for evaluating therapeutic response than standard cellular monolayers. Overall, the papers published in the Research Topic highlight the transition from simplified systems to clinically more relevant models that better represent cellular heterogeneity and morphology, intercellular and immune interactions, and thus treatment response.

In their reviews, Jassin et al. and Vincken et al. discuss the experimental platforms for studying CAR-T cell therapies and antibody-dependent cellular cytotoxicity, respectively. They emphasize the importance of selecting the appropriate models for translational research. Similarly, de Man et al. present advanced organoid-based and autologous patient-derived tissue platform for immune-modulating drug screening. These systems preserve the critical features of the tumor microenvironment, including immune cell composition and tumor heterogeneity, which allows for clinically more relevant immunotherapeutic testing.

The mechanisms of therapeutic response and resistance are presented in the studies by Senjor et al. and Vallacchi et al. Using traditional assays, 3D and microfluidic models Senjor et al. show that inhibition of the activation of cystatin F restores natural killer cell cytotoxicity in glioblastoma. Vallacchi et al. used patient-derived 3D melanoma cultures and identified SPACA6-hosted miR-99b~125a~let-7e cluster as a regulator of melanoma resistance and immunosuppression. Results from these papers show that advanced models can reveal biological mechanisms that remain hidden in simplified traditional systems.

Functional cellular models can also be used for biomarker discovery and treatment optimization. In this regard, Gole et al. investigated responses to omalizumab treatment in severe pediatric asthma. Using their basophil activation-based model they identified *RSAD2* as a candidate biomarker associated with therapeutic response. Moreover, Pei et al. showed that expansion and activation of dendritic cells enhances anti-tumor immunity and sensitizes intrahepatic cholangiocarcinoma to anti-PD-1 therapy. These papers illustrate how mechanistic studies can guide the development of more effective treatment strategies.

Rajagopal et al. and Augustin et al. focused on the translational safety assessment. Rajagopal et al. evaluated humanized mouse models using a standardized antibody reference panel for cytokine release prediction while Augustin et al. described a comprehensive *in vitro* safety framework supporting the first-in-human evaluation of a TCR-like T-cell bispecific antibody. These studies are an example of how new methodologies can help bridge the gap between preclinical assessment and clinical application.

By describing the development of human 3D skin models as an animal-free diagnostic platform for autoimmune bullous diseases, and by reporting the establishment of immunocompetent skin equivalents incorporating both CD4+ and CD8+ T cells to better model psoriasis pathophysiology and treatment responses, Huth et al. and Vahav et al. respectively, showed that development of advanced cell models goes beyond the field of oncology. Here, the universal application of advanced modeling is presented.

Finally, the review by Jazbec and Režen expands the discussion toward emerging RNA-based therapeutics. The authors discuss the potential of circular RNAs as biomarkers and therapeutics in hepatocellular carcinoma. This paper exemplifies the constant development of immune-mediated biological therapies. However, it also points out the need for development of experimental systems that can support the development and validation of such new therapies.

The research published here shows that the future of translational oncology does not depend only on the identification of new therapeutic targets, but also on the development of appropriate models. The integration of complex cellular systems, patient-derived materials, advanced imaging, functional assays, and translational safety approaches is resulting in the development of more realistic experimental platforms. This in turn facilitates rapid biomarker discovery and improved therapeutic development, and ultimately more effective »bench to bedside« transition.

We, the topic editors, would like to express our sincere gratitude to all the authors contributing to our Research Topic, the reviewers for their expert peer reviews, and the Frontiers editorial team for their support during the publication process. We hope that the presented article collection will inspire new research in preclinical modelling. In addition, we believe that this article collection will strengthen cooperation between basic, translational, and clinical researchers working to advance immune-mediated therapies.

